# Correlation between total sperm count and sperm motility and pregnancy rate in couples undergoing intrauterine insemination

**DOI:** 10.1038/s41598-020-64578-0

**Published:** 2020-05-05

**Authors:** Sebastian Findeklee, Julia Caroline Radosa, Marc Philipp Radosa, Mohamad Eid Hammadeh

**Affiliations:** 1 Saarland University Hospital, Department for Gynecology, Obstetrics and Reproductive Medicine, Kirrberger Straße 100, Building 9, 66421 Homburg, Germany; 2 University Hospital Leipzig, Department for Gynecology, Liebigstraße 20a, Building 6, 04103 Leipzig, Germany

**Keywords:** Biological models, Endocrine reproductive disorders

## Abstract

The frequency and significance of sterility is increasing due to different socio-demographic factors in the industrialized countries. At the same time, the patients’ demand for more natural and less invasive fertility treatments is increasing. The most common method used in subfertility is intrauterine insemination (IUI). Retrospectively, the data from the patients were analyzed, in which at least one insemination and a maximum of eight inseminations were performed in the last five years (observation period 01.01.2014–31.12.2018) at the Women’s University Hospital Homburg. The primary endpoint was the onset of a clinical pregnancy. Clinical pregnancy was correlated with the partner’s total sperm count (sperm density in millions), sperm concentration and motility during insemination. These three parameters were evaluated according the World Health Organization (WHO) 2010 guidelines. The results of the spermiograms were correlated with clinical pregnancy outcome. The data were examined for 138 women with sterility, in which a total of 345 inseminations were performed (median 2.5 per woman, range 8 inseminations). There was no correlation found between spermiogram parameters and pregnancy probability in any of the inseminations. After 5 inseminations no further pregnancy occurred. The present study showed no correlation between the conception probability of intrauterine insemination (IUI) and the total sperm count/concentration/motility. After the sixth IUI, we no longer found conceptions in our patient collective. Therefore, data from this study indicate that intrauterine inseminations can be performed at all severity levels of oligoasthenozoospermia. However, the treatment should be limited to five attempts.

## Introduction

Sterility is becoming an increasing issue, that one out of seven couples experience an unfulfilled desire to have children that lasts for more than a year^1^. While assisted reproduction techniques (ART) have been in the focus of scientific interest in recent decades, there are increasing numbers of studies in recent years devoted to the optimal conduct and outcome of intrauterine insemination (IUI)^2^. One reason for the recent commitment to IUI certainly has been the demand of couples with subfertility for less invasive therapies and treatments with fewer side effects. From the medical point of view, this approach is acceptable since the application of IUI enables pregnancy rates being comparable with ART treatment after correct indication. Bensdorp *et al.* found a cumulative pregnancy rate of 47% (n = 207) after six IUI cycles with controlled ovarian hyperstimulation within twelve months^3^. Previous studies have focused on the impact of hormonal stimulation and follicle size on IUI outcome and the outcome of ART. However, there is little data on the influence of spermiogram parameters on the conception probability of women undergoing fertility treatment by IUI^4–6^. Custers *et al.* investigated 391 women who received an IUI and either got up immediately afterwards or remained lying for 15 minutes^2^.

The aim of the present study was to analyze the influence of the spermiogram parameters total sperm count (sperm density in millions), sperm concentration and motility of the sperms on the success of insemination and to determine how many insemination cycles could be performed in women undergoing IUI treatment to become pregnant.

## Methods

This work was a monocentric retrospective cohort study. All women were examined and IUI was performed during a 5-year period (01.01.2014–31.12.2018) at the Women’s University Hospital Homburg. We considered the number of inseminations performed, the type of hormonal stimulation, the onset of pregnancy and the spermiogram. All IUI’s were homologous and they were performed in the morning. After sperm delivery by the partner the sperm was evaluated according to the WHO criteria. The preparation and evaluation technique for semen was performed with two layer (90, 45%) discontinuous PureSperm® lier as described earlier by Hammadeh *et al*. in 2001^7^. Briefly, PureSperm® density gradients were performed using 1 ml of each concentration into a 15 ml falcon tube. After semen liquefaction 1 ml of the ejaculate was layered on the top of each tube and centrifuged for 30 min at 300 × g. After centrifugation, the upper layer seminal plasma and 45% diluted PureSperm® was aspirated and the remaining 90% layer, which contained the selected spermatozoa, was collected from the bottom of the tubes in the new one and washed twice with IVF medium and centrifuged at 300 × g for 10 min. The pellet was re-suspended in IVF medium and was used after capacitation (1 h incubation at 37 °C, 5% CO_2_) for IUI. Sperm quality was assessed by the three parameters sperm concentration (in millions/ml), total sperm count (sperm density) and sperm motility (progressive motile and non-progressive motile in %). A distinction was made between the three categories of “below-average”, “average” and “above-average” sperm groups, according to the criteria defined by the World Health Organization (WHO) 2010^8^ (see Table 1). If one of the three parameters number, motility and density was restricted, it was by definition a restricted spermiogram. We defined the three groups “below-average”, “average” and “above average” according to the three parameters together. Sperm morphology as one of the main criteria for the assessment of sperm quality according to the WHO 2010 was not evaluated since it was documented in less than 20% of the patients undergoing IUI. Pregnancy was defined as clinical pregnancy with evidence of heart beat.

**Table 1:** Classification of the spermiogram in “below average”, “average” or “above average” according to the WHO criteria.

Spermiogram quality	Below average	Average	Above average
Total sperm count (sperm density in millions)	<33	33-46	>46
Sperm concentration (millions/ml)	<12	12-16	>16
Sperm motility (A + B in %)	<38	38-42	>42

The statistical significance was tested with the trivariate chi-square test with the aid of the statistic program SPSS version 24, IBM, Armonk, New York, USA (significance level P < 0.05).

### Compliance with ethical standards

Since spermiogram parameters and anamnestic data from patient files were evaluated retrospectively without intervention, an ethics vote could be waived after consultation with the local ethics committee of the Saarland. All methods were carried out in accordance with relevant guidelines and regulations. Informed consent for utilizing data in an anonymous form for scientific purposes was obtained from all patients (all patients were over 18) when they were admitted to the hospital.

### Ethical approval

The study was conducted in concordance to the ethical standards of the institution.

### Informed consent

Only retrospective data from patient records were analyzed without any intervention. All patients gave their agreement in analyzing and publishing data anonymously before treatment.

## Results

Retrospectively, the data of 138 patients were examined. The median age of patients performing the first insemination was 33.7 years (minimum 22 years, maximum 45 years). A total of 345 stimulated intrauterine inseminations (169 times HCG application or stimulation with HCG and clomiphene (n = 156), FSH (n = 28), FSH/LH (n = 2) or letrozole (n = 3)) were performed during this period. This corresponds to a median of 2.5 inseminations per woman. The minimum was one insemination, the maximum was eight inseminations. There were 21 pregnancies during that period, with one woman getting pregnant twice with the help of an IUI. This corresponded to a clinical pregnancy rate of 14.5%. Reasons for discontinuing the insemination treatment were the onset of pregnancy, the change of method, the transfer to another center or the abandonment of fertility treatment as a result of physical, mental or financial burdens. Six out of a total of 42 women (14.3%), whose partners had a below-average spermiogram, became pregnant by IUI. In women with partners with an average spermiogram, it was 3 out of 13 (23.1%, n = 32 inseminations) and 12 out of 83 (14.5%, n = 213 inseminations) in women with partners with an above-average spermiogram. The analysis of the individual inseminations showed no significant correlation for the first insemination (P = 0.92) or for the second (P = 0.57), third (P = 0.47), fourth (P = 0.43) and fifth (P = 0.76) insemination between the partner’s spermiogram and the woman’s conception probability. For the sixth to eighth insemination, no P-value could be calculated, since no more conceptions occurred. Table 2 gives an overview of the occurrence of pregnancies in a correlation to the examined spermiogram parameters.

**Table 2:** Probability for clinical pregnancy in the context of intrauterine inseminations depending on the spermiogram quality.

Patients	IUI 1	IUI 2	IUI 3	IUI 4	IUI 5	IUI 6	IUI 7	IUI 8
	n = 138	n = 85	n = 55	n = 34	n = 20	n = 8	n = 3	n = 2
Spermiogram below average	2/42	2/24	1/10	0/9	1/8	0/4	0/2	0/1
Spermiogram average	1/13	1/6	0/6	1/5	—	0/1	0/1	—
Spermiogram above average	5/83	3/55	1/39	2/20	1/12	0/3	—	0/1
P-value	P = 0.92	P = 0.57	P = 0.47	P = 0.43	P = 0.76	not calculated	not calculated	not calculated

## Discussion

The aim of this study was to analyze the influence of three sperm parameters (concentration, motility, density) on the IUI result. There was no correlation between spermiogram parameters analyzed and the probability of pregnancy in women who underwent an infertility treatment. After cumulative performance of five inseminations, no patient conceived, despite further treatment.

There are several explanations for our - at first sight paradoxical - observations. On the one hand, it could be that sperm processing has levelled sperm differences. Thus, increased sperm concentration can compensate for motility limitations, and conversely, improvement in motility can compensate for reduced sperm concentration. On the other hand, it could be that the women in the investigated collective had a better oocyte quality, which can also contribute to a higher conception rate despite a restricted spermiogram. For example, couples who opted for an IUI may not have had such a severe degree of sterility for so many years, and consequently, less fertility limitations. Ultimately, sterility is a multifactorial problem. Many interfering factors can modulate the conception probability (including tubal dysfunction, body mass index, lifestyle)^9^.

Consistent with previously published literature, this study showed a pregnancy rate of 14.5%. In the published literature mean pregnancy rates after insemination treatment of 10–20.5% are given^10–12^. Until the beginning of the 21st century, the IUI was the method of choice for couples with 1–2 years of existing sterility and inconspicuous gynecological and andrological diagnosis or mild forms of andrological subfertility. The NICE (National Institute for Health and Care Excellence) guidelines, which defined the more advanced methods of Assisted Reproduction Techniques (ART) as standard therapeutic methods, changed the situation in 2003^13^. Furthermore, favored by a lower subsidy by the health insurance, there was a decrease in inseminations. Some authors even suggested that IUI in idiopathic sterility had no advantage over intercourse at the optimal time^14^. However, there has been a trend towards returning to the IUI in recent years. The reasons for this are the patients’ desire for less invasive and less intrusive methods, less time or financial problems. This seems reasonable, as most couples can achieve pregnancy without medical help.

To the best of our knowledge, there are hardly any studies to date that investigate the association between conception and pregnancy probabilities through IUI and spermiogram parameters^15,16^. Among the advocates of rapid application of ART techniques to couples with subfertility, the estimated higher rate of pregnancy in these techniques is argued^17,18^. In clinical practice, the rule is that after inconspicuous diagnosis, depending on the age and temporal pressure of the couple, at least 4-6 inseminations are recommended. In part, and especially in younger couples <30 years, even more inseminations are performed. In the case of a restricted spermiogram, one should quickly switch to *in vitro* fertilization (IVF) or, if necessary, intracytoplasmic sperm injection (ICSI) after 1–2 inseminations. We can not recommend this procedure on the basis of our data. If one recommends the IUI, one can use this procedure also in couples with under-average spermiogram four to five times. However, more insemination attempts do not seem to be recommended with regard to our data and should no longer be carried out. However, in couples with oligoasthenoteratozoospermia (OAT) ° I-III, the immediate performance of an ICSI does not seem justified, as pregnancy can be achieved even with severely limited spermiogram parameters with an average probability of 15% per treatment. The findings of the present study are consistent with data from Ombelet *et al.* and Ochsenkuhn *et al*.^19,20^. Besides, we agree with Schinfeld *et al*. concluding that semen analysis parameters do not provide information about whether a man’s sperm can fertilize an egg, and the probability of that man generating a pregnancy^21^.

Nonetheless, we are aware that our study has some limitations that make it difficult to generalize the results. First, it is a retrospective study. This examines associations and correlations, but no causal connections. Due to the nature of an observational study and retrospective design, impacts of other factors on the outcome cannot be teased out.

Furthermore, we did not analyze the spermiogram parameter morphology, because it was not regular documented in the patient files and its interpretation is contradictory between WHO 2010 and strict criteria. During the last decade, the reference value was decreased from 16 to 4% which makes conclusive comparisons difficult. Besides, we cannot report about the long-term outcome of the pregnancies achieved as it is our clinic’s policy to continue patient care just until the detection of heart beat. Afterwards, the patients are sent to the assigning gynecologists.

On the other hand, sterility is a multifactorial problem in which many factors influence each other. Not all factors are open to analysis by outsiders (e.g. previous genital infections, lifestyle, oocyte quality, partnership conflicts). In our study there is also the potential for bias due to interaction between sperm parameters. For example, a high total sperm count could upgrade the probability for pregnancy for a man with low sperm motility and vice versa. Due to the monocentric character, the number of patients and inseminations is limited and the study may be underpowered. With each insemination, the number of patients continues to decrease, so that no statistical analysis is possible for the last three inseminations. Additionally, it is a known problem of all retrospective studies that data acquisition is dependent on the quality of documentation in patient files. In the case of our study, we did not find a complete documentation of the sperm parameter volume. Therefore, we could not include them into analysis. A multi-center study with more patients would be better suited to define the multifactorial interactions of each factor on IUI success. We are planning to perform a multivariate analysis to consider all factors influencing conception rate, such as age or kind of stimulation. This study solely aimed to examine the correlation between sperm quality and IUI success.

## Conclusions

In summary, the present study showed no correlation between the conception probability of IUI and the spermiogram parameters concentration, density and motility. We were unable to find conceptions in our patient collective from the sixth IUI attempt. In this respect, the recommendation of more than five inseminations according to our data is not recommended. Considering the retrospective nature, there is a need for cautious interpretation of the current results, which awaits being confirmed by further research with advanced study design and analysis.

## Data Availability

We disclose any restrictions on the availability of materials and information.
